# Exploring Skin Tone Diversity in a Plastic Surgery Resident Education Curriculum

**DOI:** 10.1177/22925503231195023

**Published:** 2023-09-06

**Authors:** Jane Zhu, Raahulan Rathagirishnan, Chantal Valiquette, Alexander Adibfar, Laura Snell

**Affiliations:** 1 Temerty Faculty of Medicine, University of Toronto, Toronto, Canada; 2 Faculty of Health Sciences, 4257 Queen's University, Kingston, Canada; 3 Division of Plastic and Reconstructive Surgery, Department of Surgery, University of Toronto, Toronto, Canada; 4 71545 Sunnybrook Health Sciences Centre, Toronto, Canada

**Keywords:** surgical education, equity, diversity, skin tone, photogrammetric analysis, analyse photogrammétrique, diversité, enseignement de la chirurgie; équité, nuances de peau

## Abstract

**Background:** Gaps remain in surgical education regarding the representation of skin tone diversity. To improve equity and prevent misdiagnosis leading to worsened health outcomes, efforts must be made to ensure educational photographs are representative of the diverse patient populations plastic surgery residents will be treated in their future practices. **Methods:** Four study investigators examined 96 h of recorded lecture seminars from a Canadian plastic surgery resident education curriculum from May 2020 to December 2021. Using Fitzpatrick skin type to codify skin tone, photographic images were individually classified and compared. Program lecturers and residents were invited to participate in an online anonymized survey to explore related perceptions of the curricula. **Results:** A total of 1990 images were included for analysis. Of these, 83.2% were Fitzpatrick types I to III, 13.1% were Fitzpatrick types IV to V, and 3.7% were Fitzpatrick type VI. There was a statistically greater proportion of Fitzpatrick I to III compared to types IV to V (*P* < .01), and type VI (*P* < .01). Fleiss’ Kappa was calculated to be 0.896, representing near-perfect agreement. In the survey, 61% (14/22) of faculty respondents believe they include enough diversity in their photographs, however, 46% (4 of 9) of resident respondents would like to see more diversity in lecturers’ photographs. **Conclusions:** There is an underrepresentation of medium (Fitzpatrick types IV-V) and dark (Fitzpatrick VI) images in plastic surgery resident educational images. Providing a curriculum that represents diverse patient populations is crucial to enabling competency and equity of care, particularly in a highly visual field. Incorporating skin tone diversity into educational curricula should be a priority for all plastic surgery programs.

## Résumé

**Historique :** La formation en chirurgie comporte des lacunes en matière de représentation de la diversité des nuances de peau. Pour accroître l’équité et éviter les mauvais diagnostics qui aggravent l’état de santé, des efforts doivent être consentis pour que les photos pédagogiques soient représentatives de la diversité des populations de patients que traiteront les résidents en chirurgie plastique dans leur future pratique. **Méthodologie :** Quatre chercheurs ont examiné 96 heures de séminaires enregistrés dans le cadre d’un programme canadien de formation des résidents en chirurgie plastique entre mai 2020 et décembre 2021. À l’aide de la classification de Fitzpatrick pour les types de peau, ils ont classé toutes les images photographiques individuellement, puis les ont comparées. Ils ont invité les professeurs et les résidents à participer à un sondage anonymisé en ligne pour explorer les perceptions à l’égard du programme. **Résultats :** Au total, 1 990 images ont été incluses dans l’analyse. De ce nombre, 83,2% faisaient partie des types I à III de la classification de Fitzpatrick, 13,1% des types IV et V et 3,7% du type VI. La proportion d’images de types I à III selon la classification de Fitzpatrick était statistiquement plus élevée que celles de types IV et V (*P* < .01) et de type VI (*P* < .01). Le kappa de Fleiss a été établi à 0,896, ce qui représente une concordance presque parfaite. Dans le sondage, 61% des professeurs répondants (14 sur 22) étaient d’avis qu’ils présentaient une assez grande diversité dans leurs photos, mais 46% des résidents répondants (quatre sur neuf) auraient aimé en voir davantage. **Conclusions :** Les nuances de peau moyennes (types IV et V selon la classification de Fitzpatrick) et foncées (type V selon la classification de Fitzpatrick) sont sous-représentées dans les images pédagogiques présentées aux résidents en chirurgie plastique. Il est essentiel de proposer un programme représentatif de la diversité des populations de patients pour garantir la compétence et l’équité des soins, particulièrement dans ce domaine hautement visuel. L’intégration de la diversité des nuances de peau au programme de formation devrait constituer une priorité pour tous les programmes de chirurgie plastique.

## Introduction

Plastic surgery is a highly visual field, and reliance on visual data is paramount in the diagnosis, planning, treatment, and follow-up of patient outcomes. Despite this, there remain disparities in the representation of racial and ethnically diverse populations in plastic surgery materials. An analysis by Massie and colleagues of over 25,000 color images published across six plastic surgery journals and the New England Journal of Medicine reveals that only 22% of images were non-White.^1^ This trend is very apparent in breast-related surgery literature, with non-White skin representing only 8% of images,^2^ and in general medical textbooks, with overrepresentation of light skin tone as seen in 74.5% of images.^2,3^ Reports from the field of dermatology, which is similarly reliant on visual recognition for diagnoses, reveal few resources providing images of lesions on darker skin despite clinical differences in the presentation of pathology.^4,5^

The paucity of skin tone diversity in educational materials carries important implications for patient outcomes. Although melanoma is more prevalent in non-Hispanic White populations, survival in Black, Asian American/Native American/Pacific Islander, and Hispanic populations is significantly lower.^6^ Racialized groups more commonly present with ulceration, advanced disease, distant disease, and thicker tumors.^7,8^ Factors leading to delayed diagnosis include differences in clinical presentation, paucity of educational materials, and a subsequent knowledge gap.^7^ Research shows that having access to images of skin cancers in patients of darker Fitzpatrick skin types results in increased diagnostic confidence.^9^ Residency training provides much of the exposure needed to make accurate clinical diagnoses; however, more than 30% of chief residents denied having didactic sessions focused on skin of color.^10^

In addition to the importance of diversity in educational materials, there is also a need to improve racial and ethnic diversity in surgery trainees and faculty to optimize patient compliance, trust, and overall outcomes. While there has been an increase in some visible minority populations among trainees in plastic and reconstructive surgery, there is substantial underrepresentation of Black and Hispanic trainees and staff in the United States,^11–13^ even despite an increase in Black medical graduates and applicants.^14^ Similar trends are seen among general surgery residents in Canada, with a recent survey revealing that 70% of residents did not identify as a visible minority.^15^

The disparities in depicting diversity in medical education materials have sparked various global initiatives. In 2020, a clinical handbook called “*Mind the Gap*” was created to highlight clinical signs and symptoms in darker skin tones.^16^ Recently, medical students at Queen's University have led efforts to improve the skin tone diversity of images shown by faculty in dermatology lectures.^17^ Similar to dermatology, plastic surgery learning is heavily reliant on images and visual representations of various conditions.

Our project seeks to build upon this advocacy work by examining photographs from the current curriculum and associated seminars at a Canadian plastic surgery residency program. While we are focusing on skin tone diversity in this project, it is important to recognize the importance of other identifying characteristics such as ethnicity, gender, and the intersectionality of these features.

## Methods

### Image Collection

Images were selected from recorded lecture seminars from the plastic surgery resident education curriculum at a Canadian institution. At our center, the seminar lectures from the plastic surgery resident education curriculum comprised the primary modality of structured teaching delivered by faculty members and were thus used as source material. Associated seminar notes typically included pictorial (ie, drawing) but not photographic images, and therefore were not included in analysis. The study was approved by the research ethics board of a large tertiary care center, and we received permission from faculty education leads to use teaching images in this study. All images between May 2020 and December 2021 were screened for inclusion in the study by two investigators. The plastic surgery resident education curriculum is based on 3 years; therefore, a half cycle of lectures was selected for review in this study. All colored images from the presentation slides of each lecture were indexed for the purposes of this study. Exclusion criteria included nonhuman subjects, illustrations, radiological images, and identifiable individuals within the department. At the time of analysis, only images with visible skin were included. This ensured that there was broad initial inclusion of images, with further distillation prior to analysis to meet the inclusion and exclusion criteria.

### Data Analysis

A photogrammetric analysis was conducted on all eligible images by four independent reviewers including two plastic surgery residents and two senior medical students. The Fitzpatrick scale, a subjective skin tone classification, was used to assess each image with skin.^18^ A Fitzpatrick number ranging from I (pale white skin) to VI (dark brown and black skin) was assigned to each image.^19^ Data were then grouped into the following categories: Fitzpatrick I to III (light), IV to V (medium), and VI (dark). These categorizations were determined based on descriptions of white (Fitzpatrick I-III) and nonwhite (Fitzpatrick IV-VI) skin found on DermNet and previous similar research classifying skin tones^1,2,20^ with a further grouping of nonwhite skin tones into Fitzpatrick IV to V and Fitzpatrick VI. Discrepancies in the Fitzpatrick type between investigators were resolved through consensus.^21^

### Survey

A survey regarding perceptions of skin tone diversity in the resident curriculum was sent out to residents and faculty of the Division of Plastic, Reconstructive and Aesthetic Surgery (PRAS). Informed consent was obtained through a detailed document outlining the project's aims and the participant's role prior to the start of the survey. The survey was developed by the research team based on relevant literature. The study was then piloted to two faculty colleagues in Dermatology and two faculty colleagues in Plastic Surgery for expert feedback. The survey was developed on an online platform, Qualtrics XM V122021 (Utah, USA), and the wording of questions was modified slightly for resident and faculty versions to better capture relevant demographic information and assess differing perceptions. For example, the statement “I believe it is difficult for lecturers to find photographs of patients with darker skin tones” was provided to residents in the survey, whereas “I find it difficult to find photographs of patients with darker skin tones” was provided to faculty. Likert scale questions were developed to assess several statements regarding diversity in photographs in lectures, and overall equity, diversity, and inclusion (EDI) opportunities in the division of PRAS. Complimentary statement responses were matched between resident and faculty surveys based on what the statement sought to identify (ie, “The faculty is committed to providing EDI initiatives” (faculty survey) and “There is commitment to provide EDI opportunities for residents” (resident survey)).

### Statistical Analysis and Interrater Reliability

Data were analyzed with SPSS V28.0.^22^ The number of images in each category was counted and characterized according to the Fitzpatrick score as described above. Standard deviation was reported for each set of lectures. Nonparametric Mann–Whitney U test was used to calculate significant differences between the means of Fitzpatrick types I to III, IV to V, and VI across all lectures. Two-tailed *t*-test was used to calculate significant differences in photographic diversity in each lecture when compared to the aggregate. All tests were calculated with a significance value of *P* < .05.

Interrater reliability was determined using Fleiss’ kappa on the basis of Landis and Koch's cutoffs.^23^ Twenty-two images which were deemed representative of the general content were randomly selected from the lecture slides, and each was used for categorization. Kappa scores were interpreted with Landis and Koch guidelines as follows: 0 to 0.2 slight agreement; 0.21 to 0.40 fair agreement; 0.41 to 0.60 moderate agreement; 0.61 to 0.80 substantial agreement; 0.81 to 1.0 almost perfect agreement.

## Results

### Image Analysis

A total of 96.25 h were analyzed across 30 lectures, resulting in 1990 images included for analysis. This represents images from all recorded seminars between May 2020 and December 2021, which comprise a half-cycle of lectures delivered in the plastic surgery education curriculum. Examples of lecture topics covered include thermal injury, skin and soft tissue, and craniofacial surgery.

Of the images included for analysis, 83.2% (1656 ± 47.5) were Fitzpatrick type I to III, 13.1% (260 ± 10.98) were Fitzpatrick type IV to V, and 3.7% (74 ± 6.48) were Fitzpatrick type VI (Supplemental Figure 1). Overall, there was a statistically significant difference between the number of images characterized as Fitzpatrick types I to III and Fitzpatrick types IV to V (*P* < .001), as well as Fitzpatrick types I to III and type VI (*P* < .001). In total, 26/30 (86.7%) lectures contained one or more Fitzpatrick type IV to V images, and 10/30 (25%) contained one or more Fitzpatrick type VI images. Fleiss’ Kappa score was calculated to be 0.896 between four interraters, representing almost perfect agreement.

The lecture topics with the greatest proportion of Fitzpatrick IV to VI images included: thermal injury (28.6%), aesthetic surgery (18.9%), and craniofacial surgery (18.7%) (Table 1). Lecture topics with the greatest proportion of Fitzpatrick Type I to III images included: skin and soft tissue (90.5%), flaps and grafts (89.3%), and trunk and lower extremity (87.4%). There was no statistically significant difference between the proportion of Fitzpatrick I to III, IV to I, or VI images in these lectures compared to the aggregate sample (see Table 1). Further exploration of the significance of these findings is outlined in the discussion below.

**Table 1.** Fitzpatrick Classification.

Lecture topic	Fitzpatrick I-III *N* (%)	Fitzpatrick IV-V *N* (%)	Fitzpatrick VI *N* (%)	*P*-value
Skin and soft tissue	486 (90.5)	48 (8.9)	3 (0.6)	.406
Thermal injury	269 (71.4)	74 (19.6)	34 (9.0)	.394
Aesthetic surgery	245 (81.1)	39 (12.9)	18 (6.0)	.377
Flaps and grafts	217 (89.3)	21 (8.6)	5 (2.1)	.312
Craniofacial surgery	191 (81.3)	32 (13.6)	12 (5.1)	.308
Hand and upper extremity	89 (83.2)	17 (15.9)	1 (0.9)	.278
Trunk and lower extremity	90 (87.4)	13 (12.6)	0	.335
Breast surgery	41 (83.7)	8 (16.3)	0	.265
Other	28 (75.7)	8 (21.6)	1 (2.7)	.262

### Survey Results

The survey was completed by 22 (22%) faculty members and 9 (35%) residents. The faculty survey was completed by 26% (*n *= 6) of female faculty and 13% (*n *= 3) of individuals that identify as a visible ethnic minority. Among residents completing the survey, 64% (*n *= 7) identified as female, and 36% (*n *= 4) identified as a visible minority. (Table 2). Sixty-one percent (*n *= 14) of faculty respondents believe they include diversity in their photographs that represent the population of their clinical practice, however, 46% (*n *= 5) of resident respondents would like to see more diversity in lecturers’ photographs. Residents do believe that it is difficult for lecturers to find photographs of patients with darker skin tones (64%, *n *= 7). Resident belief that there should be more opportunities to learn about EDI was significantly higher than faculty (*P* < .01). Finally, the majority of both residents and faculty (respectively 82%, *n *= 9; 61%, *n *= 14), believe that there is a commitment to provide more opportunities for EDI initiatives. There was no significant difference between faculty and resident beliefs that there is a commitment to provide more opportunities for EDI initiatives.

**Table 2.** Demographic Information of (a) Faculty and (b) Resident Participants in the Survey.

(2a) Faculty participants	
Characteristics	Faculty % (*n *= 22)
*Gender*	
Female	26.1 (6)
*Years in practice*	
1–10	21.7 (5)
11–20	13.0 (3)
21–30	26.1 (6)
31–40	30.4 (7)
*Visible minority*	
Yes	13.0 (3)
*Residency training location*	
Ontario	69.6 (16)
Quebec	4.3 (1)
United States	4.3 (1)
Other	13.0 (3)
(2b) Resident participants	
Characteristics	Residents % (*n *= 9)
*Gender*	
Female	63.6 (7)
*Age*	
20–30	37.3 (3)
31–40	63.6 (7)
*Visible minority*	
Yes	13.0 (3)
*Year of residency*	
Junior (R1, R2, SSTP*)	27.2 (3)
Senior (R3, R4, R5+)	63.6 (7)
*Medical school location*	
Ontario	63.6 (7)
Alberta	18.2 (2)
Manitoba	9.1 (1)

## Discussion

To our knowledge, this study is the first assessment of skin tone diversity in Canadian institution's postgraduate plastic surgery curriculum. This study found that white skin tones (Fitzpatrick I-III) were disproportionally overrepresented compared to darker skin tones (Fitzpatrick IV-VI). The methodology and findings are in keeping with the literature regarding diversity in plastic surgery educational material. A recent review by Smith et al. of the American Society of Plastic Surgeons Education Network Plastic Surgery Curriculum was conducted to assess the Fitzpatrick classification of all photographs within their teaching chapters.^24^ Most images (81.6%) were classified as lighter tones (Fitzpatrick I-II), and only 6.6% of images were of dark skin as represented by Fitzpatrick V-VI. Additionally, only 0.6% of graphics or illustrations were Fitzpatrick V to VI.^24^ Images in major North American and European journals regarding breast reconstruction and aesthetic surgery demonstrate predominantly white skin.^25^ A notable exception is reporting of blepharoplasty, a procedure that is popular in Asian populations.^26^ Finally, a study of social media platforms used to demonstrate surgical procedures for patient education concluded that there was a predominance of white skin (88.1%) in both reconstructive and aesthetic surgery images.^27^

While an imperfect proxy, skin tone is one aspect of representation of racial and ethnic diversity, particularly within an educational field which is highly visual in nature. The study findings indicate that the photographic representation of patients in educational material does not necessarily represent the ethnic and racial diversity in the Greater Toronto Area. In 2016, 52% of Toronto's population identified as belonging to a visible minority group, whereas an average of 16.7% of images used in educational materials were representative of darker skin tones (Fitzpatrick types IV-VI). Increased diversity among skin tones seen in educational imaging is essential to develop a better proportional representation of patient populations. This in turn can act as a protective factor in preventing advanced disease and delayed diagnosis.^7^

In our study, we observed a comparatively lower number of Fitzpatrick types I to III photographs in aesthetic surgery lectures, although this was not statistically significant when compared across lectures. This finding may align with previous studies that suggest increasing proportions of non-Caucasian patients undergoing cosmetic surgery.^26,28^ One study found non-Caucasian aesthetic patients in the United States grew from 23% in 2005% to 34% in 2020, and there are certain procedures (eg, blepharoplasty) which are predominantly performed in Asian populations.^26^ Some literature exists of surgeons tailoring procedures, such as rhinoplasties and buttock contouring, to address cultural norms of non-Caucasian populations and non-Western societies.^29,30^ However, although this landscape is changing, the majority (66%) of cosmetic surgery patients remain Caucasian.^28^ This disparity may contribute to decreased skin tone diversity in photographic representation for cosmetic procedures, which further leads to lower proportions of non-Caucasian patients seeking aesthetic surgery as images may not be reflective of diverse populations.

Our study also demonstrates that perceptions of skin tone diversity differ between residents and faculty. While residents believe there needs to be more photographic variety across the Fitzpatrick scale, faculty members believe there is enough inclusion of diverse photographs in their teaching material. Interestingly, there is no statistical difference in the belief that there is a commitment to provide more EDI opportunities within the curriculum. These findings suggest that both residents and faculty believe that EDI-informed educational reform is an ongoing area of improvement which may include increasing skin tone diversity among educational images. It is important to note that this research was conducted with the support of the faculty at our institution's Division of Plastic, Reconstructive, and Aesthetic Surgery. A key step in improving diversity and equity is prioritizing inclusive research and educational initiatives. Leadership support is key to the growth of any actionable initiative, particularly when there is limited literature highlighting formalized EDI education initiatives in plastic surgery.^31,32^

There are several limitations of this work. First, this study is a single-institution observational study with a limited time frame, and further work is required to investigate trends at other centers. As the Division moves to integrate EDI teaching into its curriculum,^32^ we sought to examine lectures before full integration had taken place. While the study was limited to one site, this factor enabled our analysis to be more standardized and of higher quality. In a follow-up investigation, a multi-institutional study could be beneficial to understand how different curricula incorporate skin-tone diversity. Second, the evaluation of images is inherently dependent on the reviewers and is subject to reporter bias and one's interpretation of an image and its Fitzpatrick classification. Fortunately, our evaluation of interrater reliability showed almost perfect agreement across raters. The Fitzpatrick classification's initial intended use was for the evaluation of ultraviolet light exposure and damage, not skin tone diversity.^18^ While it can serve as a reference range for skin-tone diversity, this does not always accurately correspond to one's self-reported race or ethnicity.^33^ It is therefore not an optimal measure of race as it cannot perfectly identify white skin tones versus skin tones of some visible ethnic minorities. However, it appears to be the current standard in the literature to assess diversity in medical imaging, and its use as a proxy remains useful while we await the development of new assessment tools.^34^ Along similar lines, it is possible that images may be distorted by photography settings and lighting differences, especially under operating room lighting. This may change the interpretation of skin tone by either lightening or darkening the appearance of the skin. Furthermore, only images were extracted from lectures, therefore it is possible the lack of additional context may omit discussions during lectures which promote EDI within the curricular content. Lastly, although the lectures were delivered between 2020 and 2021, it is possible that there is outdated content in the lectures which may not reflect the current Fitzpatrick representation of patients. However, our institution attempts to mitigate this by delivering lectures in cycles which are updated every 3 years by residents and faculty. In terms of the survey, nonresponse bias is also a possible limitation given unaccountable differences that may exist between those residents and faculty that completed the survey versus those that did not. Lastly, it is also important to note that while we discuss skin tone, race, and ethnicity in the context of diversity, they are not interchangeable. Race speaks to shared physical traits, such as skin tone, whereas ethnicity is indicative of shared cultural traditions, beliefs, and language.^35^

Inequities in care on the basis of race, or racialized appearance, continue to be prevalent in the Canadian healthcare system.^36,37^ Recently, a call to action by Snell et al encouraged Canadian Plastic, Reconstructive, and Aesthetic colleagues to evaluate their programs in three domains: education, disparities in clinical settings and policies, and leadership.^32^ Within the education domain, consciously curating an inclusive setting was suggested to attract the strongest future surgeons. Our study describes skin tone representation in the plastic surgery curriculum in one of Canada's most diverse, urban centers. We also present an opportunity to address gaps in visual representation by encouraging educators to diversify the images they include in their teaching. Such a change can be effectively accomplished by making small, intentional changes in lecture/seminar images by reflecting on current images and purposefully including a wider range of diverse images.^38^ Using established resources, such as “Mind the Gap” or “visualDx,” when there is difficulty obtaining one's own case images may be of additional benefit.^16,39^ Future studies may analyze skin tone diversity educational image inclusion across multiple institutions and surgical programs (ie, across national plastic surgery programs and departments of surgery) and examine differences in measured skin tone image diversity after the implementation of EDI principles into education curricula. We also hope to explore perceptions of the current curricula, unpack explicit or implicit biases, and better understand efforts being made to improve racial and ethnic diversity within the curriculum.

## Conclusion

This study highlights an underrepresentation of darker skin tones displayed in a Canadian plastic surgery resident education curriculum. Resident training is critical in the formation of future practicing plastic surgeons and providing a curriculum that represents a diverse population provides residents with a better understanding of their patients and how the same conditions may present on different skin tones. Integrating a curriculum that acknowledges EDI and implements it in training can help develop culturally competent surgeons who provide better care.

## Supplemental Material

sj-docx-1-psg-10.1177_22925503231195023 - Supplemental material for Exploring Skin Tone Diversity in a Plastic Surgery Resident Education Curriculum Supplemental material, sj-docx-1-psg-10.1177_22925503231195023 for Exploring Skin Tone Diversity in a Plastic Surgery Resident Education Curriculum by Jane Zhu, Raahulan Rathagirishnan, Chantal Valiquette, Alexander Adibfar and Laura Snell in Plastic Surgery
