# Supplementary figures and images for: Exploring Skin Tone Diversity in a Plastic Surgery Resident Education Curriculum
Source: Plast Surg (Oakv). 2023 Sep 6;33(1):172–8. doi: 10.1177/22925503231195023 (PMC11770717; doi:10.1177/22925503231195023)

Supplemental Figure 1. Image analysis


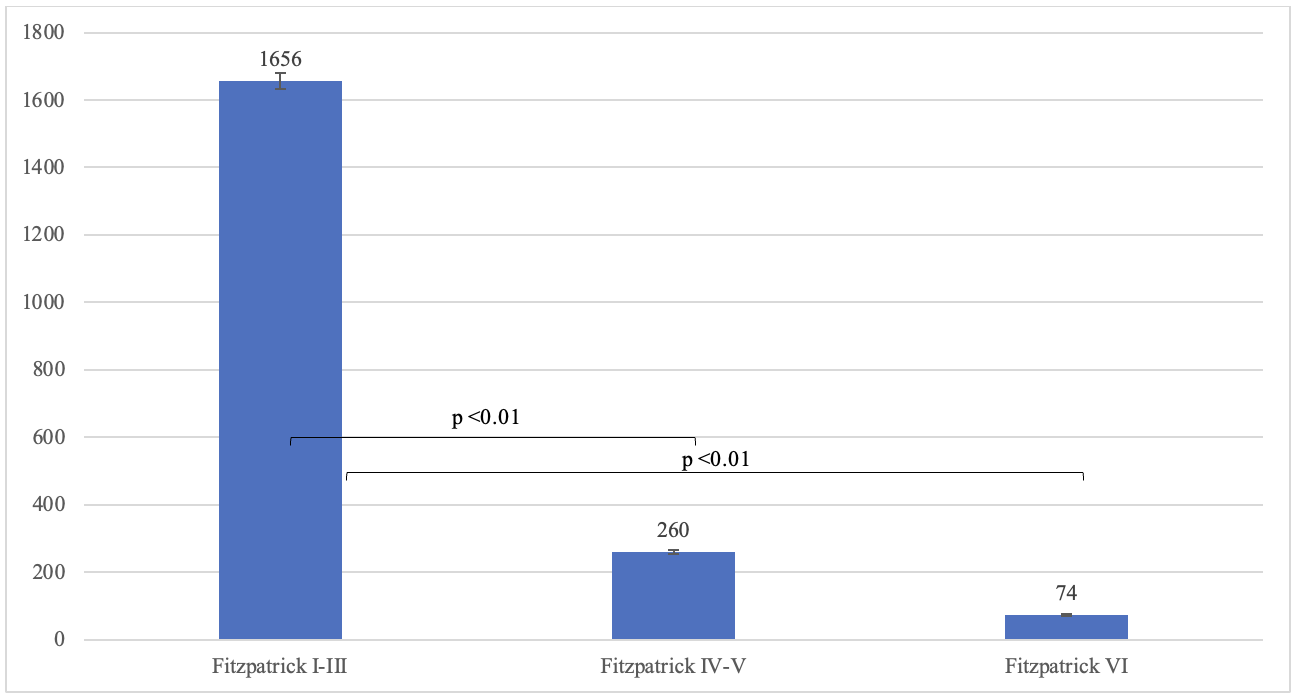

Supplement: sj-docx-1-psg-10.1177_22925503231195023 - Supplemental material for Exploring Skin Tone Diversity in a Plastic Surgery Resident Education Curriculum [file sj-docx-1-psg-10.1177_22925503231195023.docx]
